# The use of locum doctors in the NHS – results of a national survey of NHS Trusts in England

**DOI:** 10.1186/s12913-023-09830-9

**Published:** 2023-08-23

**Authors:** Gemma Stringer, Jane Ferguson, Kieran Walshe, Christos Grigoroglou, Thomas Allen, Evangelos Kontopantelis, Darren M. Ashcroft

**Affiliations:** 1 Alliance Manchester Business School, Institute for Health Policy and Organisation, The University of Manchester, Booth Street West, Manchester, M15 6PB UK; 2 Health Services Management Centre, University of Birmingham, Birmingham, UK; 3 Manchester Centre for Health Economics, Division of Population Health, Health Services Research and Primary Care, The University of Manchester, Manchester, UK; 4 Danish Centre for Health Economics, University of Southern Denmark, Odense, Denmark; 5 NIHR School for Primary Care Research, Centre for Primary Care, Division of Population Health, Health Services Research and Primary Care, The University of Manchester, Manchester, UK; 6 Division of Informatics, Imaging and Data Science, University of Manchester, Manchester, UK; 7 NIHR Greater Manchester Patient Safety Translational Research Centre, The University of Manchester, Manchester, UK; 8 Centre for Pharmacoepidemiology and Drug Safety, School of Health Sciences, Faculty of Biology, Medicine and Health, The University of Manchester, Manchester, UK

**Keywords:** Locum doctors, NHS Trusts, National survey, Quality of care, Patient safety

## Abstract

**Background:**

Locum working in healthcare organisations has benefits for individual doctors and organisations but there are concerns about the impact of locum working on continuity of care, patient safety, team function and cost. We conducted a national survey of NHS Trusts in England to explore locum work, and better understand why and where locum doctors were needed; how locum doctors were engaged, supported, perceived and managed; and any changes being made in the way locums are used.

**Methods:**

An online survey was sent to 191 NHS Trusts and 98 were returned (51%) including 66 (67%) acute hospitals, 26 (27%) mental health and six (6%) community health providers. Data was analysed using frequency tables, t-tests and correlations. Free-text responses were analysed using thematic analysis.

**Results:**

Most NHS Trusts use locums frequently and for varying lengths of time. Trusts prefer to use locums from internal locum banks but frequently rely on locum agencies. The benefits of using locums included maintaining workforce capacity and flexibility. Importantly, care provided by locums was generally viewed as the same or somewhat worse when compared to care provided by permanent doctors. The main disadvantages of using locum agencies included cost, lack of familiarity and impact on organisational development. Some respondents felt that locums could be unreliable and less likely to be invested in quality improvement. NHS Trusts were broadly unfamiliar with the national guidance from NHS England for supporting locums and there was a focus on processes like compliance checks and induction, with less focus on providing feedback and support for appraisal.

**Conclusions:**

Locum doctors provide a necessary service within NHS Trusts to maintain workforce capacity and provide patient care. There are potential issues related to the way that locums are perceived, utilised, and supported which might impact the quality of the care that they provide. Future research should consider the arrangements for locum working and the performance of locums and permanent doctors, investigating the organisation of locums in order to achieve safe and high-quality care for patients.

**Supplementary Information:**

The online version contains supplementary material available at 10.1186/s12913-023-09830-9.

## Background

Doctors working in temporary roles, often known as locums, may do so because they have greater control over the amount and type of work that they do [1–3]. Locums give organisations greater workforce flexibility and organisations that use locums can maintain workforce capacity to provide patient services. However, there are concerns that locums are an expensive resource and can have a negative impact on workload for permanent staff, patient safety and team function [4–6]. A recent survey of locums found they face barriers including lack of familiarity with work settings, instability and being treated differently to permanent staff [3]. A review of the literature suggests that the way locums doctors are engaged and used by organisations may result in some risks to patient safety [4]. A national survey of responsible officers found that there were limitations in the appraisal and revalidation processes for doctors working as locums and that locums had higher rates of deferral of revalidation compared to other doctors [7]. Furthermore, a recent qualitative study found that perceptions of locum doctors as inferior to permanently employed doctors in terms of quality, competency, and safety can result in marginalisation, stigmatisation and limited opportunities for training and development [8].

A number of reports show that in recent years the number of doctors choosing to work as locums is increasing [9, 10]. The use of locums has been linked with staff shortages, doctors leaving the profession early [11, 12] and increased sickness, workload and burnout as a result of the COVID-19 pandemic [13]. In the past it was reported that NHS Acute Trusts in England were spending significantly more on locum agency fees than on recruiting doctors to permanent positions [14]. There has been pressure on the NHS to reduce the spend on locum agencies but this is challenging given huge demands on NHS services [15]. NHS staff banks are managed by trusts and consist of a pool of healthcare professionals, including doctors, who make themselves available for shifts at Trust hospitals. A target was set to all Trusts in England to reduce agency costs by 17% for 2018/2019, this encouraged a ‘bank first’ approach to recruiting temporary staff, making the use of agencies a last resort [16]. However, the cost of agency staff has remained constant and the cost of bank staff has increased between 2017 and 2020 with a spend of over £6 billion on agency and NHS bank staff in England in 2019–20 [17].

There is some data on locum usage in NHS trusts collected by NHS Improvement [18] but this does not include why locums are needed, how their work is organised and how locum doctors are supported by NHS Trusts. The NHS England and NHS Employers guidance highlights ways to support locum doctors in providing safe provision of healthcare [19] but we do not have any information about how this guidance is used by Trusts.

The aim of this study was to seek information about locum work in NHS Trusts in order to better understand why and where locum doctors were needed; how locum doctors were engaged, supported, perceived and managed; and any changes being made in the way locums are used.

## Methods

### Questionnaire design

The survey was developed with input from stakeholders including a medical director, a research director, a senior leader in medical staffing, the chair of our Patient and Public Involvement and Engagement (PPIE) forum, a GP locum and a managing director of a locum agency. Drafts of the survey were sent out via email and stakeholders responded with comments, and where possible in depth discussions were conducted with stakeholders to gain further feedback. The research team discussed the comments received and made appropriate changes.

The study was approved by the Health Research Authority—National Research Ethics Service England, and the initial page of the survey stated that by completing the survey participants were agreeing to take part in the study.

An 89-question custom-built online open survey [20] was generated using Qualtrics software, [21]. We collected information about why locums were needed, how locums were recruited, supported, perceived and managed, how the work of locums compared to permanent doctors, experiences of locum agencies, familiarity with the NHSE guidance for supporting locums and how concerns about locums were dealt with. We also sought the views of NHS Trusts about the advantages and disadvantages of locum work and how they see locum doctor work changing in the future. A copy of the survey is provided in Additional file 1: Appendix 1.

For the purposes of this survey, we defined a locum doctor as a doctor in a temporary or fixed-term placement, engaged through a locum agency, internal locum bank or directly contracted by a healthcare organisation.

### Survey distribution

This was a survey of 191 NHS Trusts in England. Prior to distribution, we emailed Responsible Officers (ROs) to make them aware of the research and to encourage engagement. The survey was initially sent to Trust ROs in England and periodic reminders were sent to non-responders. Due to a low initial response rate, we then contacted non-responding Trusts by telephone to identify appropriate contacts at each Trust. The survey was then sent to each of the contacts provided by the Trusts, which included research and development departments, medical staffing departments and medical directors. The electronic link to the survey was active for seven months between June and December 2021 to allow Trusts the time to respond during the pandemic.

### Survey analysis

We analysed numeric and Likert scale data from survey respondents using frequency tables. Comparisons between NHS Trusts who responded to the survey and all other NHS Trusts in England were performed using t-tests. Since most survey responses were not normally distributed, non-parametric tests were used. Differences were investigated using Mann Whitney U Tests.

Three free-text questions were analysed using thematic analysis [22, 23] by three members of the research team who are experienced health services researchers. These questions asked about the advantages and disadvantages of locum agencies, the advantages and disadvantages of locums and the future of locum work. Employing an inductive approach – coding and theme development involved identifying patterns of shared meaning across responses. The written responses mostly consisted of short sentences which provided additional contextual detail to the quantitative questions. The written responses were read and re-read to become familiar with the content, notes were made of any potential codes for each question by identifying recurring words or units of meaning [24]. Responses to the three free-text questions were combined and mapped into overarching themes which encompassed the main issues highlighted in the data (a list of these themes and illustrative quotes are shown in Table 4). One further free-text question, which asked for opinions about the NHS England and Improvement guidance about supporting locum doctors, was not included in the thematic analysis as it was specific to the guidance. Illustrative comments are included to provide contextual detail to the quantitative question asking about Trusts familiarity with the guidance.

### Respondent characteristics

We surveyed a total of 191 NHS trusts and we received 98 usable responses (a response rate of 51%), of these 89 completed the whole survey and nine answered at least half or more of the questions. A further 7 incomplete responses (answering less than half of the questions) were not included in our analysis. The responses included 66 (67%) acute hospitals, 26 (27%) mental health and six (6%) community health providers suggesting that 51.5% of all acute, 55.3% of all mental health and 40% of all community trusts provided responses to the survey The survey was completed by 35 (36%) Medical Directors and/or ROs (including Deputies and Associates), 54 (55%) medical staffing (e.g. Temporary Staffing Manager, Head of Medical Workforce), three (3%) clinical staff and four (4%) other roles (e.g. Medical HR Business Partner). One respondent did not complete the question about their job role. Using data on the employment of temporary staff by NHS Trust, supplied by NHS Improvement under a bespoke data-sharing agreement, we determined no significant difference in the reported extent of locum usage between respondent and non-respondent NHS trusts [25]. Using publically available, data we found no significant difference in Care Quality Commission ratings, [26] permanent doctor FTE [27] or deprivation [28] between respondent and non-respondent NHS trusts suggesting the responses were broadly representative of NHS Trusts generally.

## Results

### The need for locums

#### How often trusts use locums

Over three quarters of Trusts always or most of the time used locums and only one Trust reported that it made no use of locums. We asked Trusts how long locums were typically engaged for at their organisation. Trusts used locums for all different engagement lengths, but locums were most frequently needed for medium-term (1–3 months) and long-term (3 months to 1 year) lengths of time, and less frequently short-term (1 week to 1 month). Acute Trusts used locums more frequently for very short (one session to under a week) and short-term lengths of time compared to mental health trusts (*p* < 0.001) and community health providers (*p* < 0.001). The majority of trusts (*n* = 45) reported increasing their locum use during the pandemic (50.6%). Thirty-five trusts (39.3%) reported that locum use has stayed the same during the pandemic and nine trusts (10.1%) reported that locum use decreased.

#### Reasons for locum use

Trusts reported the main reason for using locums was because of difficulties recruiting doctors (see Table 1). Acute Trusts needed locums to cover planned medical workforce gaps more frequently compared to community health provider trusts (*p* = 0.008), to cover absences due to short term ill-health more frequently compared to mental health providers (*p* = 0.002) and to provide additional capacity to meet demand or need more frequently compared to mental health trusts (*p* < 0.001) and community providers (*p* = 0.021).

**Table 1:** The reasons trusts need to use locums

	**Often**	**Sometimes**	**Rarely**	**Never**	**χ**^**2**^
					**Trust type**^‡^
Because of difficulties recruiting doctors	69 (71.1)	21 (21.7)	6 (6.2)	1 (1.0)	2.25
Because of difficulties retaining doctors	12 (12.4)	38 (39.2)	39 (40.2)	8 (8.3)	1.12
To cover planned medical workforce gaps e.g. maternity/paternity leave, holiday or sabbatical	28 (28.9)	52 (53.6)	15 (15.5)	2 (2.1)	6.88^*^
To cover absences due to short term ill-health	43 (44.3)	31 (32.0)	22 (22.7)	1 (1.0)	8.20^*^
To cover absences due to long-term ill-health	25 (25.8)	50 (51.6)	20 (20.6)	2 (2.1)	4.03
To provide additional capacity to meet demand or need	34 (35.1)	38 (39.2)	21 (21.7)	4 (4.1)	16.40^**^

*N* = 97. Data is presented as frequency (%). ^‡^df = 2, *p < .05, **p < .001

#### Factors important to trusts when selecting locums

Most Trusts reported that all factors (availability, experience, cost, training and familiarity) were at least moderately important when selecting a locum with greater importance placed on availability and experience and less importance placed on cost and familiarity with the organisation.

### How the need for locum doctors is met

The most frequent method for engaging locums was locum agencies. Nearly all respondents used locum agencies that were ‘framework suppliers’^1^ and three quarters of Trusts felt that locum agencies matched their needs and provided accurate information about locums. Trusts also frequently used internal locum banks and doctors who have previously worked for the organisation. The use of Doctors Direct (NHS Professionals) and digital platforms such as Locum Nest were much less frequent. Digital platforms were used significantly more by Acute Trusts compared to community providers (*p* = 0.023) and mental health providers (*p* < 0.001). Internal locum banks were used significantly more by Acute Trusts compared to community providers (*p* < 0.001) and mental health providers (*p* < 0.001).

### NHS England and Improvement guidance about supporting locums

Familiarity with the NHS England and Improvement guidance about supporting locum doctors varied across Trusts. Over half of respondents were either very or somewhat familiar but less than half of Trusts were either slightly or not at all familiar.

In free-text comments, some respondents were positive about the guidance and reported that they followed the guidance in their organisation.

*“At [name of Trust] we apply the principles of the guidance in providing our agency locum doctors with a service induction when they start from the clinical service where they are working. Prior to them starting we provide them with a welcome providing information of where the post is, access to parking along with who their contacts are whilst in post.” [Trust 31].*

Other respondents emphasised that they follow the guidance where possible or that focus is given to certain aspects of the guidance.

*“Working short term has specific challenges. We are committed to have systems in place to support the professional development and governance of the Locum doctors. We have a dedicated Medical Lead to support and supervise the practice of the Locum Drs. Particular attention is given in pre-employment checks, occupational health and induction.” [Trust 77].*

Respondents reported that the way medical staffing was organised and the cost of using locums can be barriers to implementing the guidance within their organisations.

*“The organisation does not have a temporary staffing team it also outsources the medical bank management however this is under review. Therefore best practice is not always adhered to at [Trust name].” [Trust 87].*

Others felt that the guidance was unrealistic, contradicted other guidance requirements and did not recognise differences between Trusts. It was also felt that these issues were exacerbated by current staffing pressures.

*“Its perfect world stuff, the reality is it’s the Wild West and we are desperate to get people” [Trust 27]*

*“Difficult to do when we have to provide medical care and the substantive medical workforce are demanding locum cover…they do not read the guidance VS the GMC/CQC guidance” [Trust 53].*

*“I think it's too generalised and doesn't understand that each organisation works differently and not 1 size fits all.” [Trust 25]*

Respondents commented that the guidance would benefit from being updated.

*“The guidance was put together in October 2018 so it is dated -query around some of the content around appraisals etc.” [Trust 68]*

#### Application of the guidance

We asked Trusts how frequently they followed different aspects of the guidance when locums were working in their organisation (see Table 2). Key procedures such as verifying documentation and induction were conducted more frequently (90.3% of Trusts reported always checking documentation, while 70% always provided induction) compared with, for example, providing feedback which was always provided by just 6% of Trusts. Most Trusts said they would always report concerns about locums (85%) but support with annual appraisal (16.1%) and revalidation preparation (where appropriate) (37.6%) was less frequent.

**Table 2:** Frequency of adherence to the NHS guidance about locums

		Always	Often	Sometimes	Rarely	Never	χ^2^
							Trust type^a^
When a locum doctor is placed in our organisation we…	verify documentation (e.g. GMC registration and licence to practise, HPAN, identity, language, health clearance)	84 (90.3)	7 (7.5)	1 (1.1)	0	1 (1.1)	1.78
	provide an induction to enable them to carry out the work they are being engaged to do, including access to buildings and appropriate IT systems	65 (69.9)	20 (21.5)	7 (7.5)	1 (1.1)	0	1.34
	complete an end of placement/exit report	6 (6.5)	13 (14.0)	36 (38.7)	30 (32.3)	8 (8.6)	6.87*
	provide peer/colleague feedback for the locum doctor at the end of the placement	6 (6.5)	14 (15.1)	46 (49.5)	23 (24.7)	4 (4.3)	1.07
	support the locum doctors appraisal preparation	15 (16.1)	17 (18.3)	36 (38.7)	19 (20.4)	6 (6.5)	2.74
	provide an annual appraisal for the locum doctor, if appropriate to do so (in light of the nature and duration of the placement)	28 (30.1)	17 (18.3)	19 (20.4)	20 (21.5)	9 (9.7)	9.27*
	provide access to professional development activities	22 (23.6)	22 (23.6)	34 (36.6)	15 (16.3)	0	2.74
	encourage locum doctors to attend multi-disciplinary team meetings	44 (47.3)	29 (31.2)	17 (18.3)	3 (3.2)	0	8.71*
	inform the locum doctor and locum agency or RO (where relevant) about serious untoward incidents they have been involved in (even if they are no longer employed at my organisation)	79 (85.0)	9 (9.7)	5 (5.4)	0	0	0.41
	inform the locum doctor and locum agency or RO (where relevant) about complaints they have been involved in (even if they are no longer employed at my organisation)	69 (74.2)	21 (22.6)	3 (3.2)	0	0	1.0
	support the locum doctor to engage with revalidation systems within my organisation	35 (37.6)	16 (17.2)	19 (20.4)	16 (17.2)	7 (7.5)	4.67

*N* = 93. Data is presented as frequency (%). ^a^df = 2, *p < .05, **p < .001

### Trusts experiences of locum doctors

We asked Trusts how care provided by locums compares to care provided by permanent doctors, in a number of areas (see Table 3). Overall, care provided by locums was largely viewed as about the same as care provided by permanent doctors. For example, 82.4% of participants reported that locums were the same as permanent doctors when it came to avoiding drug prescribing errors. Some Trusts reported that care was worse, in particular in relation to continuity of care (50%), but also adherence to organisational policies and guidelines (30.8%), administrative errors (30.8%), and reporting of adverse advents (28.6%). Mental health trusts were significantly more likely than Acute Trusts to report that workload for the permanent healthcare team was worse when care was provided by locums rather than permanent doctors.

**Table 3:** How care is perceived when provided by locums rather than permanent doctors

		Much better	Somewhat better	About the same	Somewhat worse	Much worse	χ^2^
							Trust type^a^
In your experience, when care is provided by locums rather than permanent doctors what effect, if any, do you think it has on the following aspects of care?	Adherence to organisational policies and guidelines (for example, prescribing guidelines)	1 (1.1)	2 (2.2)	60 (65.9)	27 (29.7)	1 (1.1)	0.32
	Providing continuity of care	1 (1.1)	1 (1.1)	44 (48.4)	42 (46.2)	3 (3.3)	3.40
	Avoiding drug prescribing errors	1 (1.1)	1 (1.1)	75 (82.4)	14 (15.4)	(0.0)	2.58
	Avoiding administrative errors	1 (1.1)	(0.0)	62 (68.1)	26 (28.6)	2 (2.2)	1.68
	Keeping clear and accurate patient notes/clinical records	1 (1.1)	2 (2.2)	73 (80.2)	14 (15.4)	1 (1.1)	1.25
	Reporting of adverse events or untoward incidents	(0.0)	2 (2.2)	63 (69.2)	22 (24.2)	4 (4.4)	0.26
	Appropriateness of referrals	(0.0)	1 (1.1)	71 (78.0)	18 (19.8)	1 (1.1)	0.05
	The functioning of the healthcare team	1 (1.1)	3 (3.0)	70 (76.9)	14 (15.4)	3 (3.3)	3.15
	Workload for permanent members of staff in the healthcare team	4 (4.0)	16 (17.6)	54 (59.3)	17 (18.7)	(0.0)	14.77**

*N* = 91. Data is presented as frequency (%). ^a^df = 2, *p < .05, **p < .001

#### Perspectives on locum doctors and locum agencies

We asked Trusts about the advantages and disadvantages of engaging locum doctors and locum agencies and how they see locum doctor work changing in the future (see Table 4). Maintaining workforce capacity and the advantage of a flexible workforce to deliver short-term and long-term service was one of the main advantages reported by respondents. Using agencies also provided assurance that compliance checks were in place; however, compliancy checks were not always done resulting in delays in locums being recruited.

**Table 4:** Perspectives on locum doctors and locum agencies

Theme	Illustrative quotes
Workforce capacity and flexibility	*“Helps us fill our gaps by accessing doctors that we do not have access to, particularly at short notice.” [Trust 19]* *“Locums provide cover to support the permanent workforce when gaps arise, which could otherwise compromise patient care and safety.” [Trust 19]* *“Advantage is a flexible workforce has helped us over the last 2-years to overcome the difficulties in staffing levels and activity.” [Trust 83]* *“Locum doctors work is flexible, it gives the doctor the chance to see many aspects of medicine without the requirement of a long term commitment, however the organisation employing the doctor in the future will need to support the doctor’s requirements and take on RO responsibilities, revalidation and appraisal *etc*.” [Trust 6]*
Compliance	*“Assurance that they have already had all appropriate background checks and connection through a single RO makes communication easier.” [Trust 46]* *“Full compliance not always complete at the time of putting the medic forward creating delays from CV approval.” [Trust 10]*
Lack of alternative options	*“No option in the current climate” [Trust 21]* *“Used as a last resort” [Trust 36]* *“exhausted all other options” [Trust 49]* *“Needs must! Not enough doctors so no choice” [Trust 39]* *“a necessary evil” [Trust 14]* *“I would like to see this become an exception to support short and immediate staffing issues and not become a reliance to solving NHS recruitment gaps” [Trust 46]* *“We would like to reduce our reliance on locums, but this is a hard slog in terms of agreeing new establishments and recruiting permanent staff” [Trust 7]* *“I would like to see all locum doctors working *via* a national NHS locum bank with fixed rates so there is no bargaining for increases in rates and playing Trusts off against each other” [Trust 42]*
Cost and control	*“They will just keep getting more expensive.” [Trust 40]* *“Becoming more expensive, working relationships worsen.” [Trust43]* *“I would like to see a shift in culture of being realistic regarding rates of pay. This can almost feel that the NHS is being held to ransom to deliver patient care and maintain patient safety. I understand this is the case regarding supply and demand due to the pandemic.” [Trust 31]* *“You need to have eyes in the back of your head and micro manage the appointment otherwise you will be overcharged on pay and commission rate. They will also cut corners on due diligence and checks.” [Trust 42]* *“Agencies drive up rates and use the top of the above capped rate as the new baseline, i.e. no one will work within the capped rates.” [Trust 54]* *“[Locum agencies] play the market and prices are always high and negotiations are time consuming and energy sapping” [Trust 55]* *“Expensive and operate a monopoly over cost as they control the flow of doctors and compete with each other for placing a doctor, offering higher rates to entice new business.” [Trust 83]* *“Agencies sometimes manipulate messages and information for their own gain, making things frustrating when we're working with tight timescales (e.g. keeping us chasing about a specific locum who will never actually start, in order for them to not lose the booking).” [Trust 44]*
Familiarity and continuity of care	*“Lack of familiarity and engagement with the department and organisation.” [Trust 16]* *“They fill the staffing gap but we would really prefer our own internal locums rather than external locums for continuity and org knowledge.” [Trust 35]* *“There is a huge difference between using an unknown doctor from an agency and using one of our own current or former doctors *via* our Medical Bank. We always prefer to use Bank for that reason. Bank is usually (but not always) cheaper.” [Trust 48]* *“Better to have a long term locum rather than occasional shifts as this allows for understanding the policies of the organisation, team work and continuity of care.” [Trust 33]* *“Would be nice to see further development of passport between Trusts, so that info can be handed over, particularly across the ICS. Would also be good to provide a shadowing / placement process for locums new to the NHS—we have concerns about taking on a locum for their first ever shift in the NHS.” [Trust 19]*
Organisational development	*“Disadvantages: when the primary driver is financial as is invariably the case, their assumptions and reason for being there is framed in their minds in that way. It does not support a developmental approach either personally or with the team. If we were to be more hospitable and offer the 'NHS' privileges of training and time for study *etc. *and all the other trust support for being a substantive member of staff, then what is the point of being within the NHS? We will have permanent staff and that is not conducive to building a stable workforce which is the requisite for workforce development, flexibility in services and the meeting of the transformation agenda. Additionally, locums are seldom interested in service improvement, do audits of any worth or take part in quality improvement. Being financially driven these are not seen as important enough. Finally, self-development is not seen as a priority which comes through reflection and a deep understanding of their practice and if not in the same place for long enough to develop good relationships that they trust to give them difficult information, this is very limited” [Trust 55]* *“The bottom line is that it inhibits the development of teams, reduces continuity of care, probably doesn't work for the locum in terms of career development, makes organisational development more difficult and is way more expensive.” [Trust 27]*
Reliability	*“Service instability due to locum doctor leaving with short notice.” [Trust 24]* *“Short term cancellations resulting in staffing gaps which could lead to patient safety risks.” [Trust 45]* *“Quality not always reliable, service provision is not always as good as that we would usually get from a substantive doctor especially at higher grades.” [Trust 62]*

In the written responses many Trusts reported they did not want to use locums, particularly due to cost, but they did not feel there was any alternative, especially given current workforce challenges. Respondents did not envisage a reduction in the use of locums due to current staffing pressures across all NHS organisations. Respondents wanted the cost of locums to reduce but did not see this happening and felt that as a consequence working relationships would deteriorate. It was felt that agencies control the market and drive up costs through competition. Costs were often above capped rates, commission rates were seen as exploitative and trust in locum agencies was low. Some respondents perceived locums as incentivised by money and some perceived that they were not invested in service improvement and self-development. Respondents felt that their organisations would like to reduce their reliance on locum agencies by increasing the use of locums from internal banks.

Respondents reported in the free-text responses that lack of familiarity with the organisation was a disadvantage and there was a preference for using internal banks rather than agencies or one-off shifts to ensure continuity of care, greater familiarity with internal systems and better investment in the organisation. The use of locums was seen as having a negative impact on organisational development and that locums were not invested or motivated to contribute to service and quality improvement. It was felt that offering better support and incentives for locums could result in further instability in the permanent workforce. To address the risk of taking on locums who have not worked at the organisation before, respondents suggested that improved communication between Trusts is required and better systems for supporting doctors in their placements.

As reported in the written responses, the quality and consistency of locums and locum agencies was sometimes perceived as unreliable. Some respondents felt that locum agencies provided quality doctors, however more respondents felt that the quality of locum doctors provided by agencies was unreliable. Locums can leave at short notice which leads to gaps and service instability and patient safety risks.

### Dealing with concerns about locums

We asked Trusts what happens when there is a low, medium or a high-level concern about a locum doctor. We defined low level concerns as: causing no harm to patients or staff and the doctor was not at any personal risk, medium level concerns have potential for serious harm to patients, staff or the doctor was at personal risk and high-level concerns are when patients, staff or the doctor had been harmed. The action that Trusts reported they would take increased depending on the severity of the concern (see Table 5). The higher the severity of the concern the more likely that the locums and locum agencies would be informed. However, not all Trusts (67.4%) would report a locum when there was a high level concern. Trusts reported that it was common for locum contracts to be ended early when there were concerns and locums to not be used again.

**Table 5:** How Trusts deal with concerns about locum doctors

		Always	Most of the time	About half the time	Sometimes	Never	**χ**^**2**^
							Trust type^a^
Low level concern	the locum doctor is informed	45 (50.6)	29 (32.6)	10 (11.2)	5 (5.6)	0 (0.0)	5.01
	reported to the locum agency	37 (41.6)	24 (27.0)	9 (10.1)	19 (21.4)	0 (0.0)	1.51
	reported to the GMC	8 (9.0)	8 (9.0)	2 (2.3)	38 (42.7)	33 (37.1)	2.86
	the locum contract is ended early	10 (11.2)	7 (7.9)	6 (6.7)	53 (59.6)	13 (14.6)	1.48
	we would not use that locum again	15 (16.9)	10 (11.2)	7 (7.9)	45 (50.6)	12 (13.5)	1.80
Medium level concern	the locum doctor is informed	66 (74.2)	21 (23.6)	1 (1.1)	1 (1.1)	0 (0.0)	4.87
	reported to the locum agency	62 (69.7)	21 (23.6)	3 (3.4)	3 (3.4)	0 (0.0)	0.24
	reported to the GMC	22 (24.7)	18 (20.2)	6 (6.7)	37 (41.6)	6 (6.7)	1.11
	the locum contract is ended early	24 (27.0)	23 (25.8)	9 (10.1)	32 (36.0)	1 (1.1)	6.00*
	we would not use that locum again	25 (28.1)	28 (31.5)	8 (9.0)	25 (28.1)	3 (3.4)	4.81
High level concern	the locum doctor is informed	83 (93.3)	6 (6.7)	0 (0.0)	0 (0.0)	0 (0.0)	0.49
	reported to the locum agency	84 (94.4)	5 (5.6)	0 (0.0)	0 (0.0)	0 (0.0)	3.01
	reported to the GMC	60 (67.4)	14 (15.7)	5 (5.6)	10 (11.2)	0 (0.0)	1.58
	the locum contract is ended early	59 (66.3)	14 (15.7)	2 (2.3)	14 (15.7)	0 (0.0)	6.59*
	we would not use that locum again	56 (62.9)	20 (22.5)	1 (1.1)	10 (11.2)	2 (2.3)	5.48

*N* = 89. Data is presented as frequency (%). ^a^df = 2, *p < .05, **p < .001

## Discussion

We found that the use of locums was an integral part of Trust working. Trusts need locums for all different lengths of engagement and very few Trusts make no use of locums. The use of locums is driven by workforce issues like recruitment, staff sickness and planned workforce gaps. It is important to note that locums come from the finite pool of doctors, and while flexibility is increased for organisations and doctors, the use of locums does not do much to alleviate doctor shortages at a national level. Trusts face challenges with recruitment of doctors, which is reflected in recruitment and retention challenges nationally [29], this results in Trusts often needing locums long-term and having to source locums mainly from agencies at high cost. We found, as others have, that there was poor awareness, ambiguity and confusion about the national guidance for locums from NHS England and who was responsible for following it [7]. Trusts focused on processes such as verifying documentation and completing induction but less was done with regard to feedback and appraisal. This is corroborated by findings from a recent survey of agency locums which recommended that organisations provide greater support for locums to obtain evidence for appraisal and revalidation [3].

Overall, care provided by locums was viewed as about the same or somewhat worse as care provided by permanent doctors, particularly in areas like adherence to organisational policies and guidelines, continuity of care and avoiding administrative errors. One reason for this could be that mistakes are more likely to be made in environments that are unfamiliar [3]. It is concerning that the majority of Trusts in our study reported using locums between one and three months and sometimes without adequate induction and support. Appropriate supervision might prevent locums getting into difficulties; however, there is little in the NHS guidance about the provision of a named supervisor or mentor for locum doctors, despite evidence to suggest locums are at greater risk of isolation and of being complained about [30] Causes of administration errors in hospitals such as inadequate communication, and local working conditions may impact more on locums who are unfamiliar with organisational systems [31]. Locum doctors may also be more likely to make mistakes if permanent staff are unable to support them due to the increased supervisory demands that may be required when locums are unfamiliar with the organisation [6] or because of negative perceptions or discrimination [3]. Yet if a serious incident occurred, not all Trusts would inform the locum agency, RO, GMC, or indeed the locum, limiting the opportunity for shared learning when things go wrong.

While some Trusts felt that continuity of care was worse when care was provided by a locum, it should be recognised that continuity of hospital care requires more than personal continuity at the patient interface. There is a collective organisational responsibility for continuity of care, and team structures and organisational systems should be considered when evaluating continuity of care [32].

In their written responses Trusts felt that lack of familiarity and high cost was a disadvantage but these were not rated as important when recruiting, suggesting Trusts are unable to prioritise familiarity and cost because of high demand to fill gaps. Respondents felt that locum agencies match their needs and provide accurate information about locums, however there were low opinions of locum agencies in relation to cost. Trusts would like to reduce the reliance on locum agencies and make greater use of internal locum banks in order to reduce costs and increase familiarity with the organisation; guidance on establishing and using banks has been produced by the NHS Workforce Alliance and should be accessed by Trusts [33, 34]. The problem of locum cost was linked to challenges in motivating permanent staff, and protecting their contractual advantages. Offering locums NHS ‘privileges’ afforded to permanent staff was perceived to be in direct conflict with building a stable workforce. This may result in the limitation of support opportunities for locums. Research has found that locums were perceived as money oriented, were treated differently than permanent staff and were often excluded from additional support processes [8].

Some respondents expressed concern that short term placement of locums did not allow for building relationships and knowledge of the systems that contributes to self and organisational development. Other research has found that permanent staff in A&E would expect temporary staff to have less vested interest in a department, particularly if they know they are not going to return [6]. Targets have already been set for Trusts to reduce their use of agency locums and increase the use of internal locum banks [18]. This allows for more regular and familiar locums which increases trust and allows for greater investment in temporary staff in the short and long term.

The results from this survey highlight the precarity of locum work. Locum contracts can be ended early following even low-level concerns and locums may have less access to communities of practice within Trusts for providing appropriate governance, leadership and support, meaning remedying performance problems may be more difficult. Similarly, Amery and Griffin (2020) found that short training rotas limited the ability of medical trainees to engage in communities of practice in order to engage in activities and form mutual relationships [35]. This may in part explain why locums are more likely to have formal complaints about them to the professional regulator than permanent doctors [9].

This is the largest survey of the use of locum doctors in the NHS. The survey had good regional coverage, although we cannot distinguish between rural or urban locations, and was completed by different types of Trust and different staff types. The response rate was relatively high for an online survey, and responding organisations appeared to be largely similar to non-respondents in various respects, however it is possible that non-respondents may differ systematically in some way. However, in securing this response rate we were unable to collect data on differences between locums who worked for different lengths of time. Locums are a heterogeneous group and locums who were employed in an organisation for short periods were likely to be different to long-term locums. In order to keep the survey to a reasonable length, we chose to ask questions about all locums rather than repeat questions for short, medium and long-term locums.

The extent to which the views and perceptions collected in the survey are useful depends partly on the representativeness of the respondents (see above) and on how insightful their perspectives are. Medical Directors and leads in medical staffing would be expected to have a good overview of locum work and systems for managing locum engagement, however we cannot be certain if all respondents, in particularly those in other roles, would have enough experience and knowledge to answer all questions accurately or how susceptible respondents were to response biases such as social desirability and acquiescent responding. In addition, each response represents the views of just one person in the organisation and may differ from those of others in the same organisation.

## Conclusions

Locum doctors are an important resource for NHS Trusts enabling them to maintain workforce capacity and provide patient services. However, there are also a number of potential issues relating to the ways that locum work is organised, the way locums are supported, and the quality of care provided by locums. In light of our findings, action that could be taken by trusts to improve quality and safety includes making more use of known and familiar doctors to minimise the use of short term placements, which is likely to be where problems arise. Internal staff banks and longer locum engagements may mitigate some problems associated with short-term placements and reduce overreliance on costly locum agencies. Where possible and appropriate, trusts should involve locums in training and development, support appraisal and revalidation, and include locums in clinical teams. Treating locums like permanent members of staff is likely to empower and enable doctors to work more effectively in the organisation. This means allowing time for familiarisation, providing proper induction, arranging access to systems, policies and procedures, and preparing for the challenges of working in unfamiliar organisations. Organisations should be aware that unfamiliar locums often cannot fulfil the whole scope of practice of a permanent member of staff, and that this can increase the workload for permanent staff, which needs to be recognised and planned for. Trusts should familiarise themselves with and follow the NHS England guidance on using locums – and pay particular attention to providing support that goes beyond the essentials of checking locum doctors’ registration, qualifications and other details. For example, trusts should provide feedback to both the locum doctor and the locum agency about locum placements, especially if there are concerns. And lastly, to facilitate learning and improve safety, it should be unacceptable for an NHS trust to terminate a locum placement because of concerns but not act on those concerns. When they are concerns, the process should be documented and the NHS trust and RO should be involved to deal with the concerns as they would for a permanent member of staff.

### Supplementary Information

**Additional file 1.** 

## Data Availability

The datasets used and analysed during the current study are available from the corresponding author on reasonable request.

## Abbreviations

NHS: National Health Service
PPIE: Patient and Public Involvement and Engagement
RO: Responsible Officer

## Footnotes

1 In April 2016, NHS Improvement mandated all trusts to procure agency staff via NHS Improvement-approved framework agreements (NHS Improvement, 2017). To be a 'framework supplier', agencies have to apply through a tender process and provide information about their organisation. Such information includes policies and procedures, an ability to supply the quality and quantity of candidates required and robust processes to meet the needs of framework customers.
