# Supplementary material for: The use of locum doctors in the NHS – results of a national survey of NHS Trusts in England
Source: BMC Health Serv Res. 2023 Aug 23;23:889. doi: 10.1186/s12913-023-09830-9 (PMC10464080; doi:10.1186/s12913-023-09830-9)
Supplement: Supplementary file 1 — Additional file 1. [file 12913_2023_9830_MOESM1_ESM.pdf]

Default Question Block

FUNDED BY NIHR | National Institute for Health Research

MANCHESTER 1824 The University of Manchester

A national survey of the use of locum doctors in the English NHS

V1 12th April 2021

This national survey about the **nature, scale and scope** of locum usage and management of locum doctors in English NHS has been funded the **National Institute for Health Research.**

We appreciate that Trusts receive many questionnaires, but the findings of this survey will provide for the first time a comprehensive dataset on the use and management of locum doctors and will provide a key input into policy-making and we therefore need your help. We are committed to using findings from this survey to inform and improve governance and working arrangements for locums. Your contributions will help inform future decisions about how locum employment is organised.

We are asking a representative, such as the **Medical Director/Responsible Officer/Medical Staffing Lead,** at all NHS Trusts in England to complete this survey. We are interested in views about why locums are needed, the governance of locums, the safety of locum working and how their work is organised by employers.

If you don't know the answer to a question please don't let that stop you from answering as much as you can. You may wish to consult with a colleague who you feel can help to answer the questions.

Ethical approval

The project has been reviewed by NHS REC (20/NW/0386) and has been given HRA approval.

Statement of Confidentiality

Everything you say in this questionnaire will remain **confidential**. There is an ID number on the questionnaire so that we know who has replied and do not send out reminders unnecessarily. To ensure anonymity please be assured **we will not report data on individual NHS Trusts**. Any information that would permit identification of an individual or a organisation will be held **strictly confidential**, will be used only for the purposes of this study, and will not be disclosed or released to other persons or used for any other purpose.

Please note that by completing the survey, you are agreeing to take part in this study.

After your responses have been entered onto a secure computer database, the survey will be securely stored in accordance with data protection regulations and destroyed after five years.

If you would like to know more about the study, please contact the research team at the University of Manchester:

Dr Jane Ferguson: jane.ferguson@manchester.ac.uk

Dr Gemma Stringer: gemma.stringer@manchester.ac.uk

The questionnaire will take around 15 minutes to complete.

You can stop any time you like and finish the questionnaire later.

Click on the arrow below to start the questionnaire.

About you and your organisation

Where do you work? [Please be assured that this survey is confidential and we will not share information or report data on individual organisations]

If your organisation is not in the list please write in

What is your job title? (select all that apply)

- ☐ Medical Director
- ☐ Medical Staffing Manager/Officer
- ☐ Responsible Officer
- ☐ ________ Other [please write in]

On an average month, approximately how many doctors are working in your organisation (excluding locums)? [Leave blank if you don't know]

____

The need for locum doctors

For the purposes of this survey we will be defining a locum doctor as: a doctor in a temporary or fixed-term placement, engaged through a locum agency / GP Chambers / locum bank / internal locum bank or directly contracted by a healthcare organisation.

How often does your organisation use locum doctors?

- ○ Never
- ○ Sometimes
- ○ About half the time
- ○ Most of the time
- ○ Always

If you never use locums, can you please explain why your organisation does not use locum doctors? [please write in]

____

How long are locums typically engaged for at your organisation?

	Often	Sometimes	Rarely	Never
very short term (single shift or under a week)	○	○	○	○
short term (one week to one month)	○	○	○	○

	Often	Sometimes	Rarely	Never
medium-term (one month to three months)	○	○	○	○
long-term (three months to one year)	○	○	○	○

What are the main reasons that you need to use locums in your organisation?

	Often	Sometimes	Rarely	Never
Because of difficulties recruiting doctors	○	○	○	○
Because of difficulties retaining doctors	○	○	○	○
To cover planned medical workforce gaps e.g. maternity/paternity leave, holiday or sabbatical	○	○	○	○
To cover absences due to short term ill-health	○	○	○	○
To cover absences due to long-term ill-health	○	○	○	○
To provide additional capacity to meet demand or need	○	○	○	○

If there are any other reasons you need to use locums in your practice please provide details.

Rate the importance of the following factors when selecting a locum.

	Extremely important	Very important	Moderately important	Slightly important	Not at all important
Availability	○	○	○	○	○
Experience	○	○	○	○	○
Cost	○	○	○	○	○
Training	○	○	○	○	○
Familiarity with the organisation	○	○	○	○	○

On average, how much does your organisation spend on locums per month? [Please be assured this information will be kept confidential, not linked to individual organisations and only used to work out an average spend on locums].

Which specialties are most likely to use locum doctors in your organisation?

Do you have any comments on why these specialties use locum doctors more than other specialties?

How is the need for locum doctors met?

How frequently do you use the following ways to engage locum doctors?

	Often	Sometimes	Rarely	Never
locum agencies	○	○	○	○
word of mouth and personal recommendations	○	○	○	○
doctors who have previously worked at the organisation	○	○	○	○
Doctors Direct (NHS Professionals)	○	○	○	○
a digital platform (such as Locum Nest)	○	○	○	○
an internal locum bank	○	○	○	○

If you use any other ways to engage locums then please provide details.

Locum Agencies

Which locum agencies do you use? [please write in]

Why do you use these specific agencies? [please write in]

Please tell us about your experiences with locum agencies

	Always	Often	Sometimes	Rarely	Never
We typically select agencies who are listed as 'framework suppliers'	○	○	○	○	○
Locum agencies provide locum doctors that match our needs	○	○	○	○	○
Locum agencies provide accurate information about locums	○	○	○	○	○

What are some of the advantages and/or disadvantages of using locum agencies?

How does your organisation use and support locums?

How familiar are you with the NHS England and Improvement guidance "Supporting organisations engaging with locums and doctors in short-term placements: A practical guide for healthcare providers, locum agencies and revalidation management services?"

- ○ Very familiar
- ○ Somewhat familiar
- ○ Slightly familiar
- ○ Not familiar at all

What do you think about the NHSEI guidance on locum doctors and how it is applied in your organisation? [Please write in]

Please tell us about what happens when locums are working in your organisation. When a locum doctor is placed in our organisation we…

	Always	Often	Sometimes	Rarely	Nev
verify documentation (e.g. GMC registration and licence to practise, HPAN, identity, language, health clearance)	○	○	○	○	○
provide an induction to enable them to carry out the work they are being engaged to do, including access to buildings and appropriate IT systems	○	○	○	○	○
complete an end of placement/exit report	○	○	○	○	○
provide peer/colleague feedback for the locum doctor at the end of the placement	○	○	○	○	○
support the locum doctor’s appraisal preparation	○	○	○	○	○
provide annual appraisal for the locum doctor, if appropriate to do so (in light of the nature and duration of the placement)	○	○	○	○	○
provide access to professional development activities	○	○	○	○	○
encourage locum doctors to attend multi-disciplinary team meetings	○	○	○	○	○
inform the locum doctor and locum agency or RO (where relevant) about serious untoward incidents they have been involved in (even if they are no longer employed at my organisation)	○	○	○	○	○
inform the locum doctor and locum agency or RO (where relevant) about complaints they have been involved in (even if they are no longer employed at my organisation)	○	○	○	○	○
support the locum doctor to engage with revalidation systems within my organisation	○	○	○	○	○

Your experience of locum doctors

How do you think locums are viewed by the following people in your organisation?

	Extremely positive	Somewhat positive	Neither positive nor negative	Somewhat negative	Extremely negative
Colleagues	○	○	○	○	○
Patients	○	○	○	○	○
Administrators	○	○	○	○	○

Please tell us about your experiences of locum doctor working and how it compares to permanently employed staff by considering the following statements:

In your experience, when care is provided by locums rather than permanent doctors what effect, if any, do you think it has on the following aspects of care?

	Much better	Somewhat better	About the same	Somewhat worse	Much worse
Adherence to organisational policies and guidelines (for example, prescribing guidelines)	○	○	○	○	○
Providing continuity of care	○	○	○	○	○
Avoiding drug prescribing errors	○	○	○	○	○
Avoiding administrative errors	○	○	○	○	○
Keeping clear and accurate patient notes/clinical records	○	○	○	○	○
Reporting of adverse events or untoward incidents	○	○	○	○	○
Appropriateness of referrals	○	○	○	○	○
The functioning of the healthcare team	○	○	○	○	○
Workload for permanent members of staff in the healthcare team	○	○	○	○	○

How you deal with concerns about locums

Please tell us about what happens when there is a low level concern (i.e. no harm to patients or staff and the doctor is not at any personal risk) about a locum doctor/s in your organisation. When there is a low level concern about a locum doctor in my organisation...

	Always	Most of the time	About half the time	Sometimes	Never
the locum doctor is informed about the concerns about them	○	○	○	○	○

	Always	Most of the time	About half the time	Sometimes	Never
the concerns are reported to the locum agency	O	O	O	O	O
the concerns are reported to the GMC	O	O	O	O	O
the locum contract is ended early	O	O	O	O	O
we would not use that locum again	O	O	O	O	O

Please tell us about what happens when there is a medium level concern (i.e. potential for serious harm to patients, staff or the doctor is at personal risk) about a locum doctor/s in your organisation. When there is a medium level concern about a locum doctor in my organisation...

	Always	Most of the time	About half the time	Sometimes	Never
the locum doctor is informed about the concerns about them	O	O	O	O	O
the concerns are reported to the locum agency	O	O	O	O	O
the concerns are reported to the GMC	O	O	O	O	O
the locum contract is ended early	O	O	O	O	O
we would not use that locum again	O	O	O	O	O

Please tell us about what happens when there is a high level concern (i.e. patients, staff or the doctor has been harmed) about a locum doctor/s in your organisation. When there is a high level concern about a locum doctor in my organisation...

	Always	Most of the time	About half the time	Sometimes	Never
the locum doctor is informed about the concerns about them	O	O	O	O	O
the concerns are reported to the locum agency	O	O	O	O	O
the concerns are reported to the GMC	O	O	O	O	O

	Always	Most of the time	About half the time	Sometimes	Never
the locum contract is ended early	○	○	○	○	○
we would not use that locum again	○	○	○	○	○

Initiatives

Are you aware of any policies or initiatives that are being used or developed by your organisation about the use of locums? [if yes, please tell us about them below]

In your opinion, what are the advantages and disadvantages of using locums? [please write in]

How do you see locum doctor working changing in the future? [please write in]

Impact of the pandemic on locum employment

How has the coronavirus pandemic impacted locum employment in your organisation?

- ○ The use of locums increased in my organisation during the pandemic
- ○ The use of locums has stayed the same in my organisation during the pandemic
- ○ The use of locums decreased in my organisation during the pandemic

Has the type of work locums typically carried out in your organisation changed during the pandemic?

- ○ Yes
- ○ No

Please provide details

Please use the text box below to provide any other details about the use of locums in your organisation during the pandemic

Final comments

Is there anything we haven't asked or anything else you would like to tell us about locum working in the NHS? [Please write in]

Thank you and submit

Thank you for your time in completing this questionnaire, your feedback is of great value to this work. If you want to make any changes to your answers then use the back button, otherwise click on the blue arrow to submit your answers.

Survey report option

If you would like a copy of the survey report then please provide your email address below. Please be assured this information will be kept confidential and your email address will not be linked to any of your answers.

Interview option

If you would like to take part in an interview about your experiences of locum practice, please provide your email address, we will be in touch to arrange an interview at a date and time that is convenient to you.

Interviews typically last around thirty minutes to an hour and can take place on Zoom/Teams/over the phone (whichever you prefer) and will be recorded with your permission.

Please be assured this information will be kept confidential and your email address will not be linked to any of your answers.

Powered by Qualtrics
